# An In Vitro Evaluation of Periotest Implant Stability Measurements Taken on Implant Retained Crowns and Healing Abutments

**DOI:** 10.1002/cre2.910

**Published:** 2024-06-16

**Authors:** Cianna O'Brien, David Naughton, Bahman Honari, Lewis Winning, Ioannis Polyzois

**Affiliations:** ^1^ Department of Restorative Dentistry and Periodontology, Dublin Dental University Hospital University of Dublin, Trinity College Dublin Ireland; ^2^ Department of Biostatistics, Dublin Dental University Hospital University of Dublin, Trinity College Dublin Ireland

**Keywords:** dental implants, perio test value

## Abstract

**Objective:**

To assess the reliability of implant stability measurements recorded with the Periotest device and to investigate the differences in values when these measurements were taken on implant retained crowns and healing abutments.

**Materials and Methods:**

Fifty‐six implants in eight synthetic bone blocks were used to carry out implant stability measurements using the Periotest device by two different operators. Each block constituted an example of bone of density D1, D2, D3, or D4, and two blocks of each density were used. The healing abutments placed were of a height to allow approximately 6 mm of the implant‐abutment complex to be supracrestal and temporary crowns were made to match the dimensions of an average central incisor. Descriptive statistics were used to describe the perio test values (PTVs) at each of the different heights on the implant abutments and implant crowns. Means for each site were calculated and distribution of data assessed using the Kruskal Wallis test. The interclass correlation coefficient (ICC) was used to determine the relationship between the PTVs recorded on the implant abutments and implant crowns.

**Results:**

The mean PTV (±standard devidation) recorded across all sites was 5.57 ± 11.643 on the implant abutments, and 12.27 ± 11.735 on the temporary crowns. Excellent/good inter‐operator ICCs were recorded for the mid‐abutment site in all bone blocks D1–D4 (ICC = 0.814, *p* < 0.001, ICC = 0.922, *p* < 0.001, ICC = 0.938, *p* < 0.001, ICC = 776, *p* < 0.001). For mid crown sites, ICC between operators was excellent/good only for recordings in D2 bone (ICC = 0.897, *p* < 0.001).

**Conclusions:**

Periotest device seems to be able to reliably measure implant stability across all types of bones when the implant stability is assessed at approximately 3 mm coronal to the implant platform for abutments and 4.5 mm for implant supported single crowns.

## 1 Introduction

The assessment of implant stability is essentially an assessment of the implant‐bone interface and the percentage of bone‐to‐implant contact present to hold the implant in a fixed position (Alsaadi et al. 2007). Therefore, factors such as bone density (Alsaadi et al. 2007; Truhlar et al. 1997), implant length, and surface characteristics of the implant (Ochi, Morris, and Winkler 1994), and the patient's healing capacity have an impact on the implant stability that can be achieved (Aparicio, Lang, and Rangert 2006). It is important for both clinical and research practice to be able to assess the implant stability objectively.

Resonance frequency analysis (RFA) is a technique developed for clinical measurement of implant stability and osseointegration. It is determined by the stiffness of the bone‐implant interface and the distance from the transducer to the first bone‐implant contact (Meredith 1998). The prototype instruments gave the results in Hz, however by the time the first commercial version of the RFA technique (Osstell, Integration Diagnostic AB, Goteberg, Sweden) became available the results were expressed as the implant stability quotient (ISQ) (Aparicio, Lang, and Rangert 2006). The implant stability quotient ranges in value from 1 to 100, where a high ISQ value indicates high implant stability and a low ISQ value indicates low stability. The ISQ values are based on the stiffness of the implant bone system and the calibrated resonance frequency of the transducer used (Turkyilmaz et al. 2009; Aparicio, Lang, and Rangert 2006).

Meredith (1998) showed that the vertical position of the implant, the marginal bone level and the abutment height all influenced the resonance frequency. Clinical limitations of the RFA technique include the fact that it cannot be used on implants with cemented restorations or implants that are no longer in production (Lachmann et al. 2006). The orientation at which the transducer is held in relation to the implant has also been shown to have an impact on the ISQ recorded, where Fischer et al. (2008) found that mesial‐distal measurements had higher RFA values recorded than buccal‐palatal ones for all implants.

The Periotest (Gulden‐Medizintechnik, Benscheim an der Bergstrasse, Germany) was originally developed by Schulte et al. (1983) in order to dynamically measure the reaction of the periodontium to a defined impact load (Olivé and Aparicio 1990; Aparicio, Lang, and Rangert 2006). The periotest value (PTV) indicates periodontal damping capacity and was designed to assess tooth mobility. It has since been used also to assess implant stability, however the range is narrower owing to the fact that a stable implant with an implant‐bone interface has more stiffness than a tooth surrounded by periodontal ligament (Aparicio, Lang, and Rangert 2006). The range of the Periotest device is from −8 to +50, with −8 being most stable and +50 being the least stable. In a preliminary study, Olivé and Aparicio (1990) determined that the normative range for implants might lie between –5 and +5 PTV. It is understood that the vertical positioning of the implant, the abutment height, the level of the marginal bone and the striking position of the rod on the implant or implant abutment are critical factors for accuracy and reproducibility of results (Aparicio, Lang, and Rangert 2006). The clinical limitations of the Periotest device include the greater measurement error when used in vivo compared to in vitro experiments (Meredith 1998, Reynolds, Winning, and Polyzois 2023). The different superstructures and their attachment mode has also been shown to have an impact on the PTV result (Gomez‐Roman and Lukas 2001). Furthermore, as demonstrated by Meredith (1998) there is an increase in PTV by 1.5 units for each millimeter away from the marginal bone. Therefore, the superstructure upon which the reading is made has a significant impact on the resultant PTV. The manufacturers also recommend that the Periotest handpiece be positioned in a particular manner in order to obtain valid and reproducible readings.

This in vitro study aimed to assess the reliability of implant stability measurements recorded with the Periotest device and to investigate the differences in values when these measurements were taken on implant retained crowns and healing abutments. Based on existing literature and the number of articles published using the Osstell device for measuring implant stability, RFA would generally be considered the gold standard device. As a result, we used the RFA measurements recorded by Naughton, Donnelly‐Swift, and Polyzois (2023) for the same cohort of implants as a control against which the PTVs measurements would be compared.

The optimal position for placement of the Periotest hand‐piece on the implant abutment and crowns was also assessed. Finally, we sought to determine differences in implant stability for different implant systems and their possible effects on the Periotest measurements.

## 2 Materials and Methods

This study involved the use of the Periotest device to record the damping capacity of a number of implants placed in synthetic bone blocks of varying density. An apparatus was employed to support the Periotest handpiece at different levels for each implant on each block. As this is an in vitro study of nonbiological materials it did not require ethics approval.

The seven implants placed on each bone block were:

1. Standard 4.1 × 10 mm SLA (Institute Straumann AG, Basel, Switzerland)
2. Standard Plus 4.1 × 10 mm SLA (Institute Straumann AG, Basel, Switzerland)
3. Tapered Effect 4.1 × 10 mm SLA implant (Institute Straumann AG, Basel, Switzerland)
4. Standard 4.8 × 10 mm SLA (Institute Straumann AG, Basel, Switzerland)
5. BNST 4.0 × 10 mm internal hexagonal connection implant (Zimmer Biomet, Barcelona, Spain)
6. BOET 4.0 × 10 mm external hexagonal connection (Zimmer Biomet, Barcelona, Spain)
7. Ankylos C/X 3.5 × 11 mm (Dentsply Sirona, Hanau, Germany).

The synthetic bone blocks were composed of resin polyurethane (BoneModels, Castellón de la Plana, Spain) and each individual block constituted an example of bone of density D1, D2, D3 or D4 as described by Lekholm and Zarb (1985). Two blocks of each density were used, resulting in a total of eight bone blocks. Each bone block had seven implants placed at uniform distance from each other according to the manufacturer's protocols. The osteotomies in the D1 bone blocks required the use of a bone tap prior to implant placement, while the osteotomies in the D4 synthetic bone blocks were undersized to enable placement of an implant that was stable. Therefore, there were a total of 56 implants used in this study. All implants were placed according to the implant manufacturers' protocols using the official method for each system drills.

**Figure 1:**
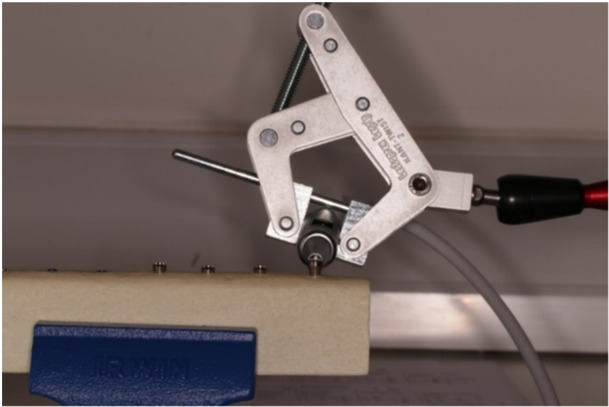
Articulated gauging arm (Fisso, Strato Line Model: S‐20 Arm [length, *L*, 200 mm)] connected to a clamp [KT2 Alu quick clamp] which held the Periotest handpiece in position.

The apparatus to support the Periotest pen was an articulated gauging arm (Fisso, Strato Line Model: S‐20 Arm [length, *L*, 200 mm]) sourced from MAPRA Technik (Figure 1). The Periotest machine used was the Periotest classic (Medizintechnik Gulden e.K. Eschenweg 3, 64397 Modautal, Germany).

A number of healing abutments and temporary crowns were used in this study.

The healing abutments used were:

- Straumann 4 mm × 4.5 mm
- Straumann 4 × 3 mm
- Zimmer Biomet® EP one piece 4.1 × 4 × 6 mm (internal hexagonal connection)
- Zimmer Biomet® EP one piece 4.1 × 4 × 6 mm (external hexagonal connection)
- Ankylos® standard C/sulcus former—6 mm height.

The healing abutments were matched to the implant manufacture type and of a height to allow approximately 6 mm of the implant‐abutment complex to be supracrestal to the synthetic bone blocks. The temporary crowns were manufactured in‐house using Elos Accurate Tibase and PMMA milled crowns, all made to the same to the dimensions of an average central incisor (width 9 mm and length 12 mm). The healing abutments and crowns were all torqued to 5 N cm using a calibrated torque wrench (Tohnichi). The National Metrology Laboratory (National Standards Authority of Ireland) calibrated the device.

As the supra crestal implant abutment complex was always the same for the abutments, the sites chosen were the most coronal aspect, the mid‐point of the abutment and the implant‐head so the measurement points were approximately 2 mm apart. For crowns, the sites chosen were the implant head and the rest of the sites were approximately 3 mm apart moving coronally. Taking into consideration that the width of the metal rod of the Periotest hand piece measures 2 mm in diameter, these distances are estimates.

The bone blocks were immobilized on a benchtop using a standard benchtop vice. The Periotest classic machine and Fisso articulated arm were set up alongside, such that the handpiece of the Periotest could be positioned in relation to each implant at a distance of 0.6–2.5 mm to facilitate a reading of the PTV of each implant to be taken.

A distance of 1–1.5 mm was chosen for the study protocol and a 1–1.5 mm acrylic block was used to standardize the distance in the study proper. These measurements were taken in triplicate by two different operators (C.O.B. and I.P.), and the mean PTV for each site was recorded in an Excel (v16.55, Microsoft Corporation, 2021) spreadsheet. Therefore, while a total of 2016 PTVs were measured, 672 of these inform the data analysis.

### 2.1 Statistical Analysis

Descriptive statistics were used to describe the PTVs at different heights in the implant abutments and implant crowns. Frequency distributions and boxplots were used to display the data. The means for each site were calculated, and the distribution of the data was assessed using the Kruskal Wallis test. The ICC was used to determine the relationship between the PTVs recorded on the implant abutments and implant crowns. Further analysis involved plotting Blant Altman plots and the Wilcoxon Signed‐Rank test. “Investigative statistics” such as Spearman's rank test, were used to assess the relationship between the PTVs recorded on implant abutments and ISQ values recorded for the same implants by Naughton, Donnelly‐Swift, and Polyzois (2023). Descriptive statistics were also used to assess the impact of the bone density and implant type on the PTVs recorded. The ICC between operators was also calculated. Moderation analysis was used to investigate the effect of bone density and implant type on the implant stability measurements recorded. Assessment of the agreement between PTV and ISQ values in determining implant stability for this cohort of implants was completed using a scatter plot that facilitated analysis of the data for all bone types, and also when D4 bone was excluded.

## 3 Results

**Figure 2:**
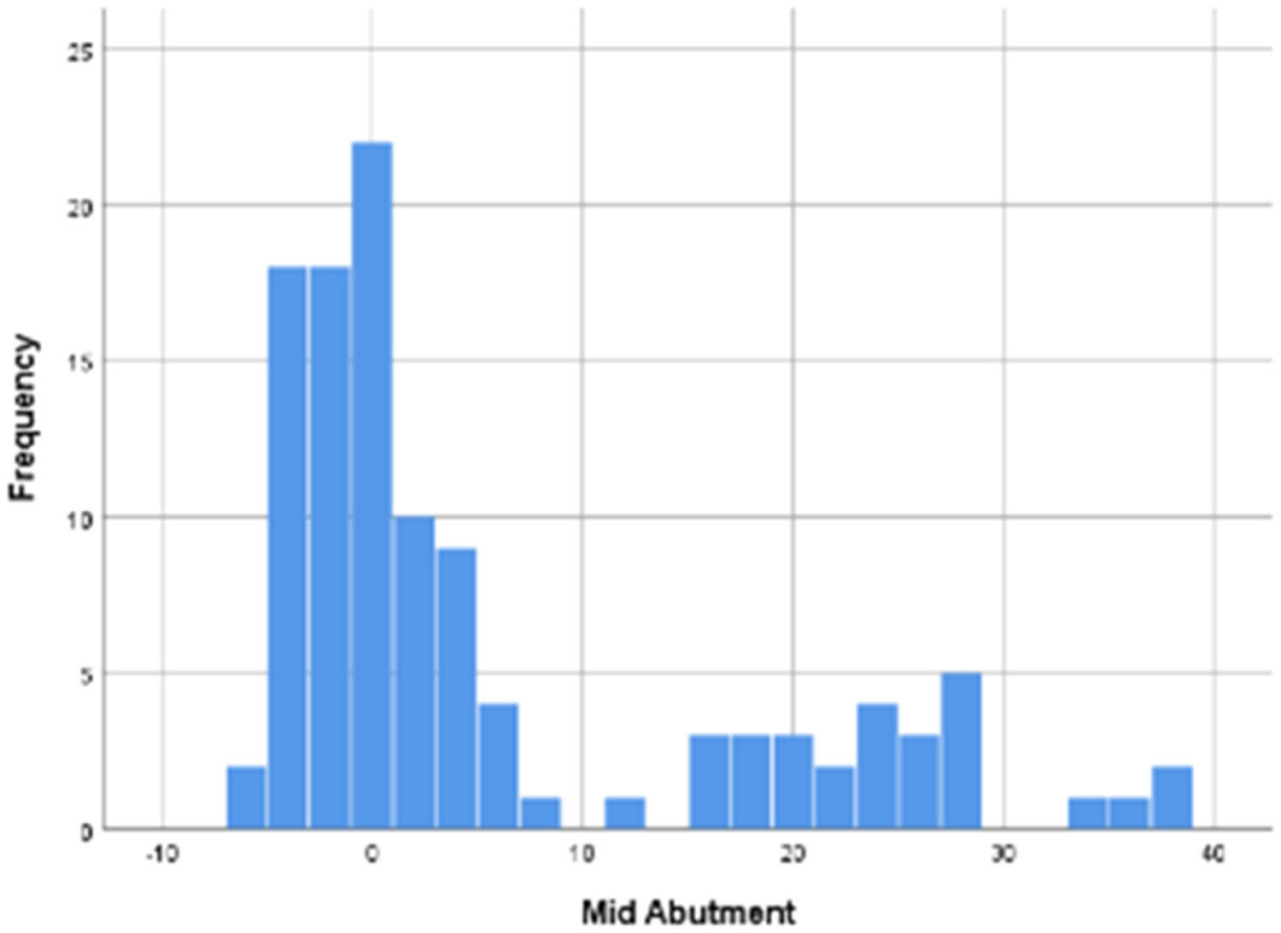
Frequency distribution of mean perio test value measured at mid of implant abutments torqued to 5 N cm.

**Figure 3:**
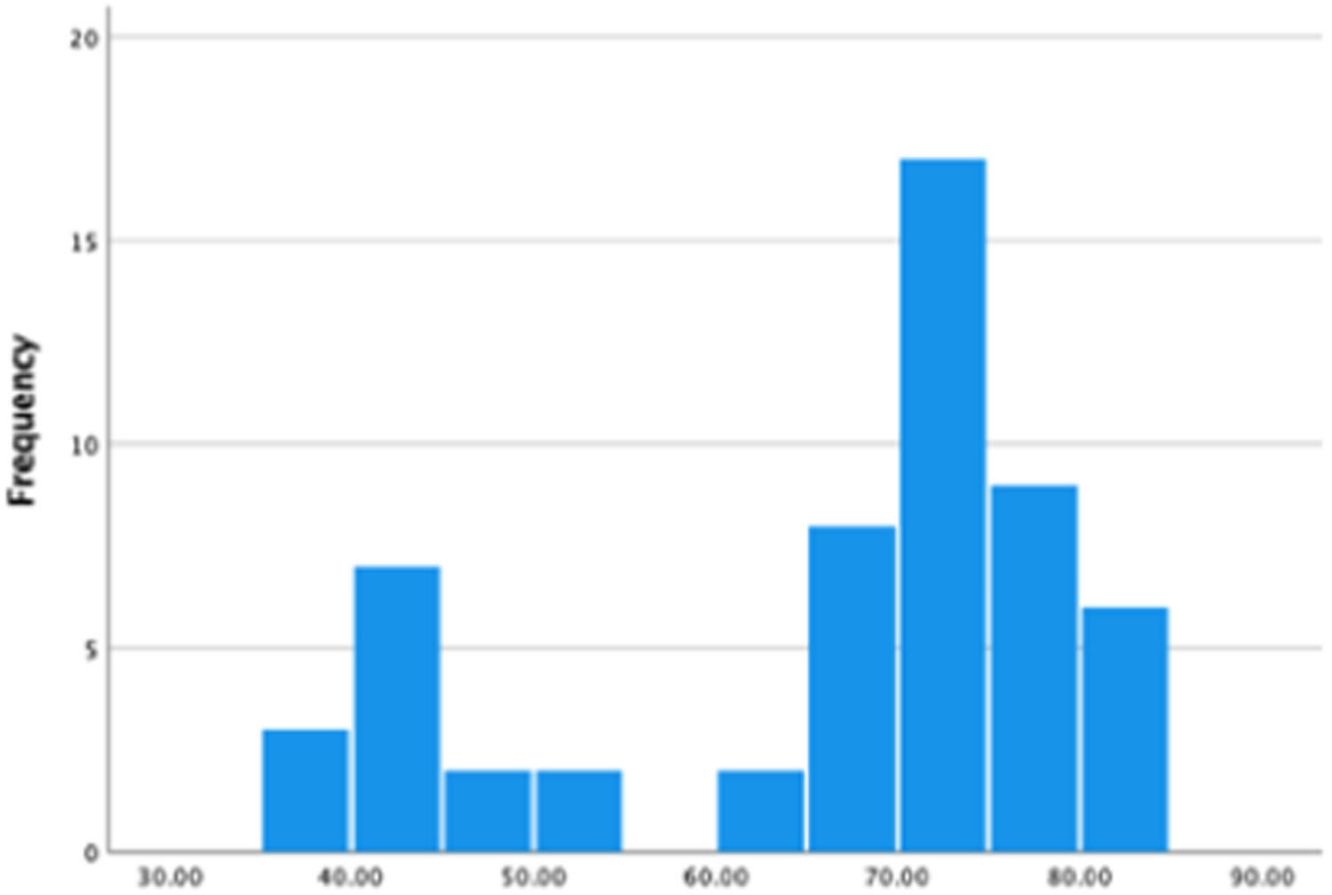
Frequency distribution of mean implant stability quotient values for implant abutment assessed with Osstell device when torqued to 6 N cm (Naughton, Donnelly‐Swift, and Polyzois 2023).

Seven implants in eight bone blocks (*n* = 56 implants) were used to carry out implant stability measurements by using the Periotest (PTV) device. The mean (±standard deviation) PTV recorded across all sites was 5.57 ± 11.643 on the implant abutments, and 12.27 ± 11.735 on the temporary crowns. The mean PTVs recorded by Periotest at the three different areas of the healing abutments and crowns can be seen in Tables 1 and 2. The distribution of the PTVs recorded at the coronal, mid‐ and implant‐head on the implant abutments and implant crowns were all similar (Figure 2). The ISQ values recorded previously by Naughton, Donnelly‐Swift, and Polyzois (2023) for the same 56 implants, present the reverse distribution (Figure 3).

**Table 1:** Mean PTVs recorded by Periotest in three different areas of the healing abutments.

PTV	Mean, SD
Coronal abutment	7.12 ± 11.831
Mid abutment	5.32 ± 11.493
Implant‐head abutment	4.28 ± 11.526

**Table 2:** Mean PTVs recorded by Periotest at on the three different areas of the temporary crowns.

PTV	Mean, SD
Coronal crown	14.32 ± 11.829
Mid crown	11.96 ± 12.264
Implant‐head crown	10.54 ± 10.860

The inclusion of implants placed in bone blocks of D4 density accounts for the outliers of low stability. The PTV data shows a positive skew, while the ISQ data shows a negative skew. The nonnormal distribution of the PTVs recorded on the abutments and crowns placed in bone blocks D1–D4 was assessed and confirmed with the Kolmogorov–Smirnov test which had a significance value of <0.001. The results of Naughton, Donnelly‐Swift, and Polyzois (2023) demonstrate that the ISQ values recorded on implants across all bone types were also not of normal distribution. When tested with the Kolmogorov–Smirnov test the significance was <0.001.

The relationship between the ISQ values and the PTVs recorded for each site on the implant abutments was assessed using Spearman's rank correlation coefficient. A statistically significant negative correlation was found between the ISQ data and the PTVs recorded on the coronal abutment (*r*_*s*_ = −0.305, *p* = 0.022), and the mid‐abutment (*r*_*s*_ = −0.482, *p* < 0.001), but not to the implant‐head abutment readings (*r*_*s*_ = −0.232, *p* = 0.085).

The correlation between the ISQ values and the mid abutment PTV value is of moderate strength and from this, we concluded that the mid‐abutment position should be used to gain the most accurate measurement when using the Periotest device to assess the stability of an implant.

To assess the impact of using the Periotest device on an implant crown instead of an abutment to assess the implant stability, an ICC was calculated for the mid‐abutment to each site on the implant‐retained temporary crowns. The ICC was moderate for the coronal crown, and good for the mid‐crown and implant‐head crown sites. The ICC for the coronal crown was lowest at 0.700, for the mid‐crown was 0.810, and highest for the implant‐head crown was 0.847. All results were statistically significant (*p* < 0.001).

**Figure 4:**
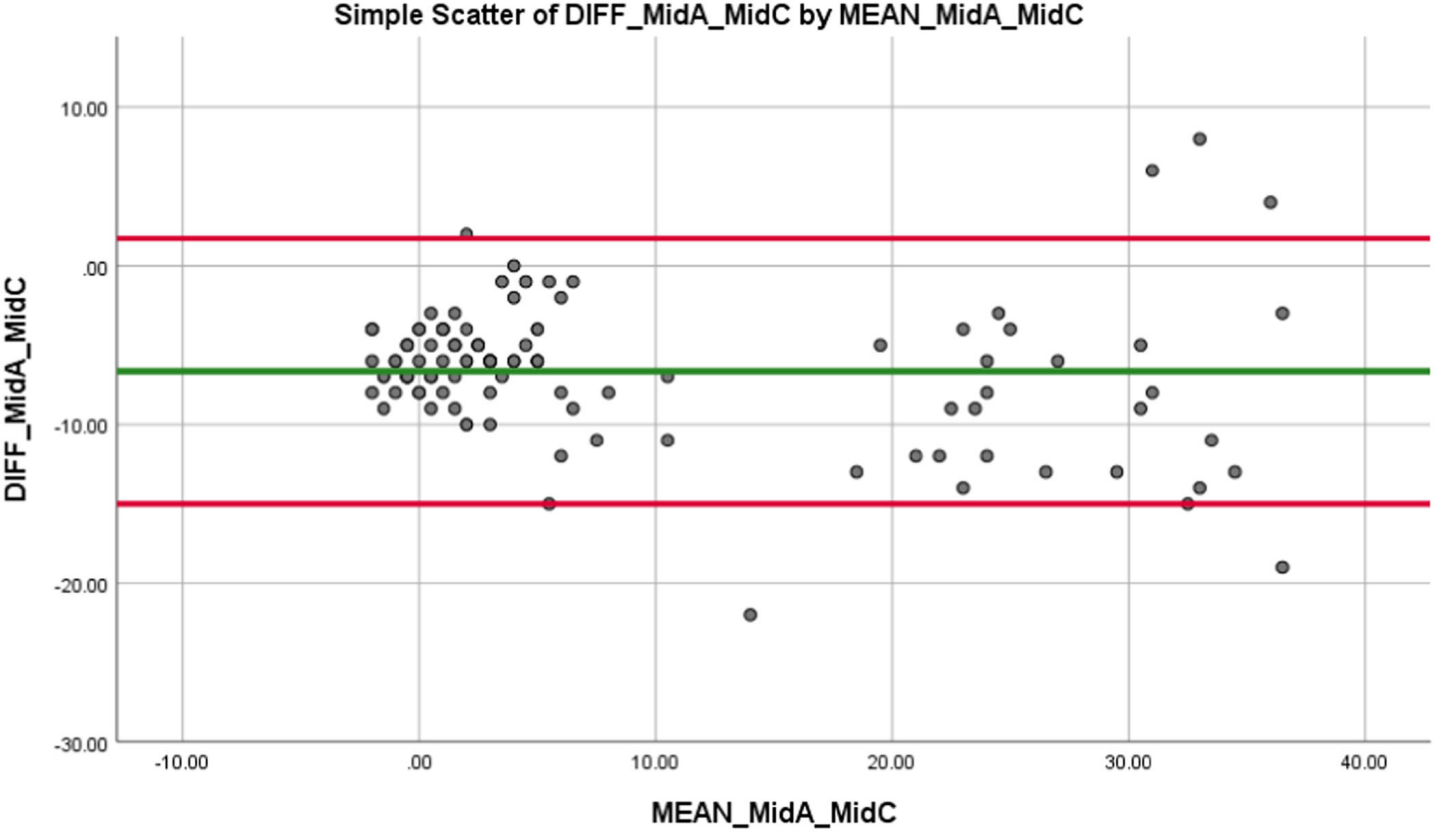
Blant Altman plot for difference versus mean of mid‐abutment versus mid crown in all bone densities.

The difference between the intraclass correlation coefficient for the mid‐crown and implant‐head crown to the mid‐abutment was negligible (0.810 vs. 0.847). Taking measurements at the implant head position on implant‐retained crowns can be extremely challenging for the clinician owing to the presence of soft tissue and the emergence profile of the restoration. Owing to the negligible difference between the ICC for the mid and head positions, and the ease with which mid‐crown measurements can be taken, the mid‐crown is of more interest to researchers and clinicians in establishing a correlation between the PTVs recorded on implant‐retained crowns and implant abutments. The Blant Altman Plot (Figure 4) displays the distribution of the difference versus the mean for the mid‐abutment to mid‐crown.

When the distribution of the PTVs recorded at mid‐abutment and mid‐crown were individually assessed using the Kolmogorov–Smirnov test, a non‐normal distribution was detected for both the mid‐abutment and mid‐crown PTVs (*p* < 0.001). Further analysis using the Wilcoxon Signed‐Rank test showed that there was a statistically significant difference between the PTVs recorded at the mid‐abutment level when compared to the mid‐aspect of the crown (*Z* = 8.715, *p* < 0.001).

It would appear that for all implant types assessed in this study across all bone types, there is a statistically significant difference between the PTVs recorded on crowns when compared to the mid‐abutment reading.

Finally, the intraclass correlation coefficient between operators 1 and 2 was calculated. The ICC between operators was good to excellent when measurements were recorded with the Periotest device on the implant mid‐abutment site for all bone types. Excellent inter‐operator intraclass correlation coefficients were recorded for the mid‐abutment site for D2 and D3 bone (ICC = 0.922, *p* < 0.001, ICC = 0.938, *p* < 0.001, respectively), and good inter‐operator intraclass correlation coefficients were recorded for D1 and D4 bone (ICC = 0.814, *p* < 0.001, ICC = 776, *p* < 0.001, respectively). This differs to the results of Naughton, Donnelly‐Swift, and Polyzois (2023) where excellent inter‐operator intraclass correlation coefficients were reported for measurements taken using the Osstell device for D1–D3 bone, but not for D4 bone. In fact, the inter‐operator ICC reported for the ISQ values recorded in D4 bone was very poor (ICC = 0.039) (Table 3).

**Table 3:** ICC between operators when measurements were recorded using the Osstell device and the Periotest device at the mid‐crown and mid abutment site of the implants across all bone types.

Bone density	ICC mid‐abutment PTV values	ICC mid‐crown PTV values	ICC ISQ values (Naughton, Donnelly‐Swift, and Polyzois 2023)
D1	0.814 *p* < 0.001	0.494, *p* = 0.020	0.944, *p* < 0.001
D2	0.922, *p* < 0.001	0.897, *p* < 0.001	0.983, *p* < 0.001
D3	0.938, *p* < 0.001	0.361, *p* = 0.094	0.803, *p* < 0.001
D4	0.776, *p* < 0.001	0.675, *p* = 0.004	0.039, *p* = 0.410

The ICC between operators was somewhat more mixed when measurements were recorded on the mid‐crown site of the implants. While the ICC between operators was excellent for recordings in D2 bone (ICC = 0.897, *p* < 0.001) and moderate for D4 bone (ICC = 0.675, *p* = 0.004), the ICC for D1 and D3 bone was poor (ICC = 0.494, *p* = 0.020) and (ICC = 0.360, *p* = 0.094), respectively (Table 3).

As the ICC for both the ISQ values and mid‐abutment PTVs recorded in D4 bone density were worse than those recorded in D1–D3 bone, it is of interest to analyze this dataset using the implant stability measurements recorded in D1–D3 bone only.

### 3.1 D1–D3 Bone Density Only

The mean PTV recorded across all sites when the D4 bone was excluded was −0.82 ± 3.050 on the implant abutments, and 5.36 ± 3.843 on the temporary crowns. The standard deviations recorded for the means when D4 bone is excluded are narrower, and as expected, the mean stability is higher. The highest mean value was at the coronal aspect (lowest stability) and the lowest mean value (highest stability) was at the implant head. This was true for measurements taken on both abutments and crowns. (Tables 4 and 5). Fewer outliers were observed in the distribution of the PTVs recorded at each site on the implant abutments and temporary crowns when D4 bone was excluded. The ISQ values recorded previously by Naughton, Donnelly‐Swift, and Polyzois (2023) also show fewer outliers and the inverse of the distribution presented by the PTVs again.

**Table 4:** Mean PTVs recorded by Periotest at different heights on implant abutments in D1–D3 bone density.

PTV site (D1–D3 bone)	Mean, SD
Coronal abutment	0.74 ± 3.359
Mid abutment	−0.82 ± 3.050
Implant‐head abutment	−1.85 ± 3.153

**Table 5:** Mean PTVs recorded by Periotest at different heights on temporary crowns in D1–D3 bone density.

PTV Site (D1–D3 bone)	Mean, SD
Coronal crown	8.18 ± 4.327
Mid crown	5.36 ± 3.843
Implant‐head crown	4.82 ± 3.761

Analysis of the distribution of the PTVs and ISQs recorded in D1–D3 bone only was carried out using the Kolmogorov–Smirnov test, which demonstrated a Gaussian distribution for the PTVs and ISQs recorded when D4 bone was excluded (*p* > 0.001).

Spearman's rank correlation coefficient was used to assess the relationship between the ISQ readings with the PTVs recorded at the different sites on the implant abutment in D1–D3 bone only. The results show that the ISQ readings have a significant moderate negative correlation with the coronal abutment (*r*_*s*_ = −0.537, *p* < 0.001), the mid‐abutment (*r*_*s*_ = −0.685, *p* < 0.001), and the head of the implant abutment (*r*_*s*_ = −0.508, *p* = 0.001). The Spearman's rank correlation coefficient test showed that the best correlation to the ISQ values recorded by Naughton, Donnelly‐Swift, and Polyzois (2023) was found for the PTVs recorded at the mid‐abutment site (*r*_*s*_ = −0.685, *p* < 0.001).

**Figure 5:**
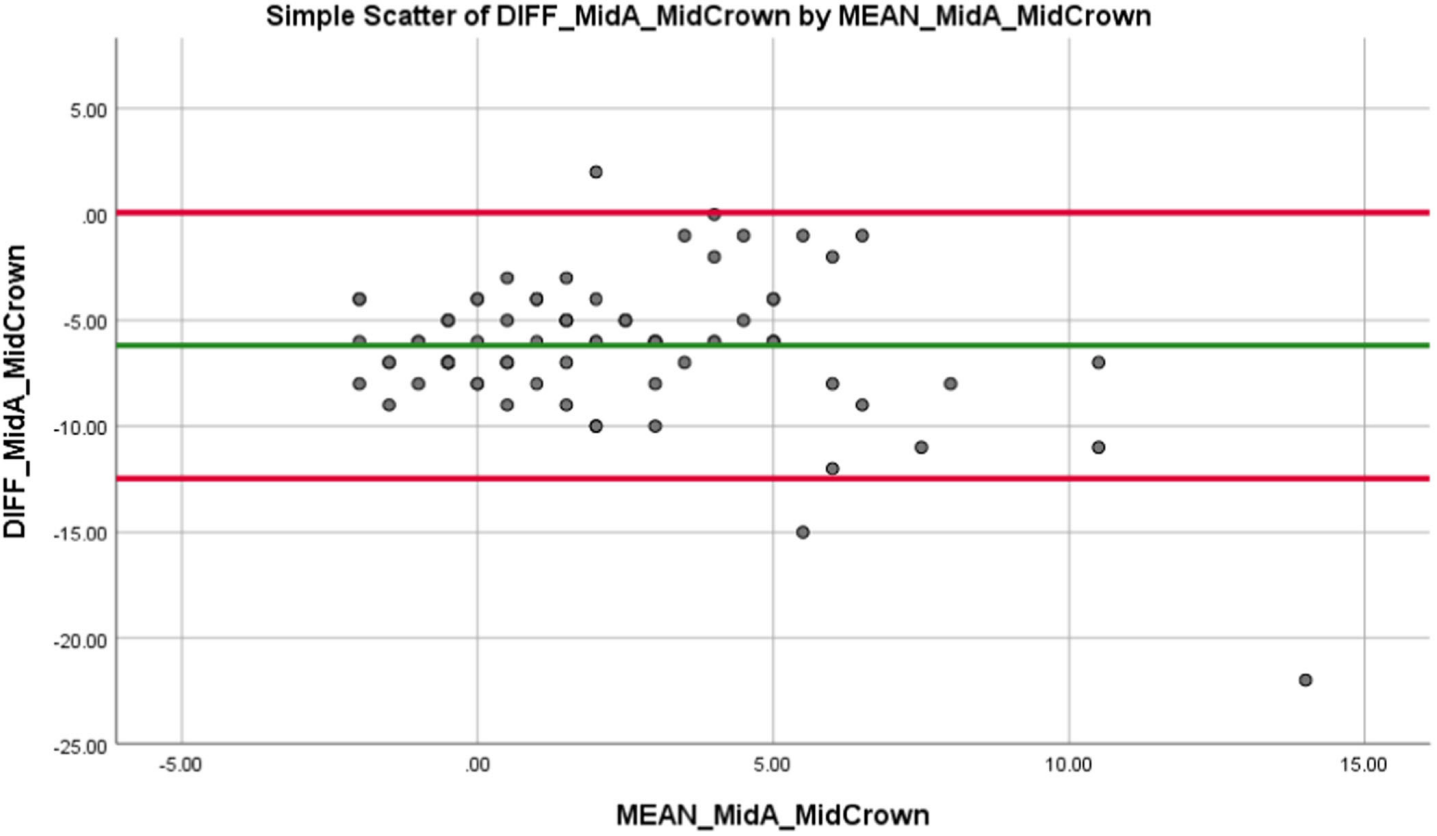
Blant Altman plot for difference versus mean of mid‐abutment versus mid‐crown in D1–D3 bone density.

The ICC was then assessed for the mid‐abutment PTVs to the different sites on the temporary crowns when D4 bone was excluded. The results show a generally poor correlation between the PTVs recorded at the mid‐abutment site and all sites on the temporary crowns in D1–D3 bone density. The ICC for mid‐abutment to the different sites on the temporary crowns was much lower when D4 bone was excluded. The highest ICC was between the mid‐abutment PTV and the mid‐crown PTV (ICC = 0.221, *p* < 0.001) when compared to the ICC for the coronal crown (ICC = 0.138, *p* < 0.001) or the head crown (ICC = 0.212, *p* < 0.001). This differs to the results recorded for the intraclass correlation coefficient calculated across all bone densities where the ICC between mid‐abutment and mid‐crown was 0.810 (*p* < 0.001). The Blant Altman plot (Figure 5) displays the distribution of the difference versus the mean for the mid‐abutment to mid‐crown when D4 bone is excluded. The majority of points lie within one standard deviation of the mean value.

Further analysis using the Wilcoxon Signed‐Rank test showed that there was a statistically significant difference when D4 bone density was excluded in the PTVs recorded at the mid‐abutment level compared to the mid‐aspect of the crown (*Z* = 7.907, *p* < 0.001). This finding is similar to the result of the Wilcoxon Signed Rank test when the PTVs recorded at the mid‐abutment and mid‐crown were compared across all bone types, resulting in a significant difference (*Z* = 8.715, *p* < 0.001). While the Wilcoxon Signed‐Rank test demonstrates that there is significant difference between the PTVs recorded at the mid‐abutment and mid‐crown sites across all bone densities and when D4 bone is excluded, further calculations are required to determine the difference in PTVs that the clinician should expect.

The impact of excluding D4 bone when analyzing the difference between the mid‐abutment and mid‐crown PTV was analyzed. This was required prior to defining a set value for the difference in PTV that clinicians should expect when implant stability measurements are recorded on an implant abutment compared to an implant crown. An independent sample test compared the mean PTVs recorded across all bone densities and when D4 bone was excluded. The results demonstrated that PTVs did not differ significantly based on whether D4 bone was included or not when assessed at both the mid‐abutment sites (*p* = 0.439) and mid‐crown sites (*p* = 0.421). Therefore, although there was a difference in the mean values reported when D4 bone was excluded compared to all bone densities, the independent sample test demonstrated that the inclusion of values recorded in D4 bone did not have a significant impact on the differences.

The difference in implant stability recorded at the mid‐abutment site compared to when the measurement was taken at the mid‐crown site was found to be 6.63 ± 4.27 PTV, when all bone densities were included. The difference between the mid‐abutment and mid‐crown sites were also assessed when D4 bone was excluded, and was found to be 6.20 ± 3.20 PTVs. Therefore, when assessing the stability of an implant with the Periotest device, the clinician should expect a difference of approximately six PTVs between measurements taken on an abutment and measurements taken on a crown, when the mid‐points of each are used. This translates to the PTV recorded on an implant crown being approximately six PTVs higher than the PTV recorded on the implant abutment, at the same time, for the same implant.

### 3.2 Impact of Type of Implant

A one‐way‐ANOVA with post‐hoc Tukey test was carried out for the implant types across all bone densities. There were no statistically significant differences detected in the mid‐abutment readings between the different types of implants when all bone densities were included. This is similar to the findings of Naughton, Donnelly‐Swift, and Polyzois (2023) where there was no statistically significant difference in the ISQ values recorded for the different implants when all bone densities were included (*p* = 0.361).

### 3.3 Association Between Mid‐Crown and Mid‐Abutment PTVs

The effect of the implant and the bone density on the correlation between mid‐abutment and mid‐crown was assessed using moderation analysis. This allowed examination of whether the variables of bone density and implant type changes the strength of the relationship between the mid‐abutment and mid crown PTVs.

The correlation between the mid‐abutment and mid‐crown PTVs were then assessed using the Pearson test and the correlation was found to be excellent (*r* = 0.954, *p* < 0.001). The relationship between the mid‐abutment and mid‐crown was examined using moderation analysis with bone as the moderator, which resulted in a statistically significant result (*p* = 0.041).

Further analysis of these results was carried out using the Pearson test to assess the relationship between the mid‐abutment and mid‐crown based on each individual type of bone density. The correlation between the mid‐abutment and mid‐crown changes based on the bone density, with a significant correlation for D2 and D4 bone, and a non‐significant correlation for D1 and D3 (Table 6).

**Table 6:** Relationship between bone density and mid‐abutment and mid‐crown PTVs.

Bone density	Relationship between mid‐abutment PTV and mid‐crown PTV as assessed using the Pearson test
D1	*R* = 0.404, *p* = 0.152
D2	*R* = 0.857, *p* < 0.001
D3	*R* = 0.237, *p* = 0.414
D4	*R* = 0.586, *p* = 0.028

Analysis of the relationship between the mid‐abutment and mid‐crown PTVs based on the impact of the implant was also assessed using moderation analysis, with the implant set as the moderator. The moderation analysis found that that the implant had no significant impact on the relationship between the mid‐abutment and mid‐crown PTV differences (*p* = 0.814). This result indicates that the type of implant does not have an effect on the difference in PTVs that can be expected when implant stability measurements are taken on the implant abutment versus on the implant‐retained crown (Table 7).

**Table 7:** Relationship between mid‐abutment and mid‐crown PTV based on implant type.

Implant type	Relationship between mid‐abutment PTV and mid‐crown PTV as assessed using the Pearson test
1	*R* = 0.966, *p* < 0.001
2	*R* = 0.986, *p* < 0.001
3	*R* = 0.996, *p* < 0.001
4	*R* = 0.995, *p* < 0.001
5	*R* = 0.931, *p* = 0.001
6	*R* = 0.987, *p* < 0.001
7	*R* = 0.947, *p* < 0.001

### 3.4 Investigation into Correlation Between PTVs and ISQ Values

The effect of the implant and the bone density on the correlation between PTVs and ISQs values was also assessed using moderation analysis. This allowed examination of whether the variables of bone density and implant type changes the strength of the relationship between the mid‐abutment PTV and the ISQ values for the same implants.

The correlation between the PTVs and ISQ values were then assessed using the Pearson test and the correlation was found to be excellent (*r* = 0.912, *p* < 0.001). The relationship between the PTVs and ISQ values was examined using moderation analysis with bone as the moderator. The results demonstrated that there was no significant impact of the type of bone density on the relationship between the PTVs and ISQ values recorded for the same implants (*p* = 0.063), when all bone types were included in the analysis. However, the moderation effect is significant at a significance level of 10% (i.e., where the *p* value is set to ≤0.10 instead of ≤0.05).

When the impact of each type of bone density on the relationship between the PTVs and the ISQ values recorded was assessed, the moderation analysis showed that D1 (*r* = −0.731, *p* = 0.003) and D2 (*r* = −0.716, *p* = 0.004) bone types showed different/significant correlations between PTVs and ISQs. However, D3 (*r* = −0.166, *p* = 0.569) and D4 (*r* = 0.265, *p* = 0.359) bone densities did not.

Analysis of the relationship between the PTVs and the ISQ values based on the impact of the implant type was also assessed using moderation analysis, with the implant set as the moderator. The moderation analysis found that that the implant had no significant impact on the relationship between PTVs and ISQ values recorded (*p* = 0.745). This result indicates that the type of implant does not have an effect on the relationship between PTVs and ISQ values (Table 8).

**Table 8:** Relationship between PTV and ISQ values as assessed using the Pearson test.

Implant type	Relationship between mid‐abutment PTV and ISQ as assessed using the Pearson test
1	*R* = −0.990, *p* < 0.001
2	*R* = −0.991, *p* < 0.001
3	*R* = −0.999, *p* < 0.001
4	*R* = −0.993, *p* < 0.001
5	*R* = −0.938, *p* = 0.001
6	*R* = −0.981, *p* < 0.001
7	*R* = −0.969, *p* < 0.001

## 4 Discussion

One putative advantage that the Periotest device might have over competitors, is that for single implants, the implant crown might not need to be removed to facilitate implant stability assessment (Bilhan et al. 2015). Therefore, one of the aims of our study included assessment of the positioning of the Periotest handpiece on implant‐supported crowns. While positioning of the Periotest handpiece is sporadically reported in detail, few studies have analyzed differences in PTVs based on the type of suprastructure and the impact this may have on the PTV recorded. A clinical study by Gomez‐Roman and Lukas (2001) investigated the differences in PTVs recorded based on whether the implant stability was assessed on a sulcus former, a crown abutment, or a single crown. The authors reported that “the Periotest measurement was performed at the center of the visible labial or buccal surface of the gingiva former, crown abutment or crown.” The results of their study demonstrated a difference between the PTVs recorded on the abutments and crowns at the same time‐point. The average individual change between the single crown and the sulcus former was −3.5 PTVs, and between the crown abutment and the single crown was −1.7 PTVs (Gomez‐Roman and Lukas 2001). The implants used in their study were Frialit‐2 implants (Friadent, GmbH). As this was a clinical study where the bone quality and quantity were not controlled, the mean differences in recorded PTVs may be interpreted with caution. Furthermore, while measurements were taken at the center of the gingival sulcus former, crown abutment or single crown, these components all significantly vary in height which may have had an impact on average individual change for each implant. Faulkner et al. (2001) demonstrated that variation in height of 1 mm can alter the implant stability measurement by 1–2 PTV. Further discrepancies may have occurred owing to different torque values used for each of the components, where the authors reported that the healing abutment was torqued to 8 N cm and the crown was torqued to 18 N cm (Gomez‐Roman and Lukas 2001). The authors concluded that the different superstructures and their attachment mode had an impact on the PTV result (Gomez‐Roman and Lukas 2001).

In the study herein, where the mid‐abutment PTV had been found to have the closest correlation with the resonance frequency analysis results, the correlation between the mid‐abutment PTV and the PTVs recorded at the different sites on the implant‐supported crowns was explored. The ICC for the mid‐abutment to the coronal crown was 0.700, while the ICC for the mid‐abutment to the mid‐crown and implant‐head crown were 0.810 and 0.847, respectively (*p* < 0.001). The difference between the mid‐crown and mid‐abutment results was considered very small, and owing to the clinical difficulties that can be anticipated from attempting to measure implant stability juxta‐gingivally, the mid‐crown site is to be recommended for implant stability assessment with the Periotest device.

Further investigation of the relationship between the mid‐abutment PTV and mid‐crown PTV naturally developed as consistent differences were recorded. This was to be expected, as Gomez‐Roman and Lukas (2001) had previously shown that the type of abutment had an impact on the PTV reported for a given implant at the same time point. The authors highlighted the importance of standardized measurements in order to reliably assess implant stability. Correlation of the relationship between PTVs recorded on the abutment to those recorded on the crown for the same implant would allow the clinician better information when analyzing implant stability changes at the different stages between implant placement and restoration.

We deduced that analysis of the above information could be used to provide a guide for clinician's as to what difference in PTVs to expect when assessing implant stability using an implant abutment vs on an implant crown, with all other factors being equal.

Khalaila, Nasser, and Ormianer (2020) had previously shown that the Periotest device was a reliable tool for assessment of implant stability and predictive bone level changes around implants. The PTVs recorded at baseline and at follow‐up correlated significantly with the bone loss detected at follow up. However, for implants with a cement retained restoration where placement of a standard abutment is challenging, the clinician may be uncertain as to whether higher PTV values recorded on crowns versus those previously recorded on abutments is due to marginal bone loss or simply on account of the longer lever. Cemented implant restorations may be used for esthetic reasons (Palmer, Palmer, and Newton 2003), or in situations of expected high occlusal load as they have been shown to have greater resistance to high loads than screw‐retained restorations (Cicciu et al. 2014).

Regarding the better ICC for D2 bone compared to D1 bone, it is of note that the Periotest device was originally designed to assess tooth mobility and the damping capacity of the periodontium, and was therefore designed to work with some flexibility in the apparatus being assessed owing to the presence of the periodontal ligament and physiological tooth mobility (Lukas and Schulte 1990). D1 bone is composed of a thick plate of cortical bone with little trabecular, while D2 bone is composed of a dense trabecular structure with a moderate cortical plate (Lekholm and Zarb 1985). Hsu et al. (2013) demonstrated that the PTVs recorded for foam blocks of the same elastic modulus was influenced by increasing the thickness of the cortical plate, where the thicker cortical plate gave a lower PTV indicating increased implant stability.

In D4 bone, the Periotest device demonstrated good ICC values between operators (D4 ICC = 0.776, *p* < 0.001). Therefore, implant stability assessed in D4 bone shows good inter‐operator reliability and reproducibility, indicating that the Periotest device functions well in D4 bone density. This is in stark contrast to the results of Naughton, Donnelly‐Swift, and Polyzois (2023) where an extremely poor inter‐operator ICC of 0.039 was found when implant stability was assessed in D4 bone using the Osstell device. The lack of cortical bone in the D4 bone might explain the findings. Similar findings were previously reported by Hsu et al. (2013) where the ITV and PTV were found to be more accurate than the ISQ values when assessing implant stability in osteoporotic bone.

An early finding in the analysis of the results of this research, was the significant discrepancy between the PTVs recorded in D4 bone compared to all other bone densities. This was true for implant stability measurements recorded on the implant abutments and implant‐retained temporary crowns. In this regard, the results of this investigation mimicked those of the study by Naughton, Donnelly‐Swift, and Polyzois (2023) using the same implant and bone block cohort.

D4 bone is not commonly encountered by the implant surgeon. Truhlar et al. (1997) found that that D4 bone was rarely encountered in the mandible, and was present in the maxilla in <20% of cases when placing over 4000 implants between 1996 and 1997. Surgeons commonly alter their osteotomy preparation when placing in D4 bone, typically by undersizing the osteotomy or by using osteotomes to enhance the primary stability (Cavallaro, Greenstein, and Greenstein 2009). In accordance with this, the implant osteotomies performed in the D4 bone blocks in this study were undersized. For the reasons listed above, it was deemed of interest in this investigation to exclude the measurements recorded in D4 bone blocks and continue analysis using the implant stability measurements recorded in D1–D3 bone density only.

Exclusion of D4 bone from the analysis led to interesting results. First, the distribution of the data now followed a Gaussian curve. This was demonstrated in the Q–Q plots and by assessment with the Kolmogorov–Smirnov test. The normal distribution of the PTVs recorded at implant abutments and implant‐retained temporary crowns when D4 bone was excluded, was similar to the previous results of Naughton, Donnelly‐Swift, and Polyzois (2023) where the ISQ values also followed a normal distribution when D4 bone was excluded.

In the study herein, variations in the implant stability achieved for the different types of implants were analyzed using a one‐way ANOVA. No variation in PTVs was detected when the implants were analyzed with all types of bone density included. This is in line with the findings of Naughton, Donnelly‐Swift, and Polyzois (2023) who did not detect any differences in implant stability based on implant type when assessing this cohort of implants with the Osstell device.

However, when the implant stability measurements recorded on implants in D4 bone were excluded from the analysis, significant differences were detected for the implant stability measurements obtained for different implants with both the Periotest and Osstell devices (*p* < 0.001).

Where our study aimed to assess the reliability of the Periotest device, we used different implant systems to see if their anatomical and connection differences would significantly affect the correlations between the ISQ and the PTV measurements as well as between the PTV's taken on crowns and abutments. No significant effects were detected. This is an important finding as nowadays, hundreds of different implant systems are in circulation. As a result, it is important for clinicians to know that the instrument they use measures what was designed to measure regardless of the type of implant examined.

Limitations of this study include the inability to include both implant type and bone as moderators when assessing the relationship between PTVs and ISQ values, and mid‐abutment to mid‐crown PTVs due to insufficient power.

Another limitation was the inclusion of only two operators. Although a calibrated torque wrench was used to torque each implant abutment and crown, inclusion of more operators would allow better assessment of the reliability of the device.

A 6 mm healing abutment was chosen to facilitate implant stability to be measured at different heights with the Periotest device, while the crowns were of approximately 12 mm height. Differences in the height will have had an impact on the PTVs recorded for the crowns compared the abutments (Chai, Yamada, and Pang 1993; Haas et al. 1995). However, discrepancies between the height, width and shape of both abutments and crowns are common in clinical practice, and impossible to standardize.

## 5 Conclusions

Within the limitations of this study, it seems that the Periotest device can reliably measure the implant stability across all types of bone when the implant stability is assessed at approximately 3 mm coronal to the implant platform for abutments and 4.5 mm for implant supported single crowns.

Implant stability measurement appear to be more reliable when measured at healing abutments than at implant supported single crowns.

Clinicians should take PTV measurements both with the abutment and the crown at baseline. This way, they have a reference PTV number for comparison at future review appointments. In cases where crown removal can become problematic, the difference of six PTVs in the relationship between the implant abutments and implant crowns can act as a guide for clinicians in the longitudinal follow up of their implants.

Finally, it seems that the anatomy of the implant and the nature of their connection, doesn't negatively affect the reliability of the Periotest measurements.

## Author Contributions

Ioannis Polyzois, Cianna O'Brien and Lewis Winning contributed to the conception and design of the study. Cianna O'Brien organized the database. Bahman Honari performed the statistical analysis. Cianna O'Brien wrote the first draft of the manuscript. Ioannis Polyzois, David Naughton and Bahman Honari wrote sections of the manuscripts. All authors contributed to the article and approved the submitted version.

## Conflicts of Interest

The authors declare no conflicts of interest.

## Data Availability

The raw data supporting the conclusions of this article are available by the corresponding author upon reasonable request.
